# Papaya Fruit Pulp and Resulting Lactic Fermented Pulp Exert Antiviral Activity against Zika Virus

**DOI:** 10.3390/microorganisms8091257

**Published:** 2020-08-20

**Authors:** Juliano G. Haddad, Victoria Carcauzon, Omar El Kalamouni, Philippe Desprès, Cyrielle Garcia, Fabienne Remize, Chaker El Kalamouni

**Affiliations:** 1 Unité Mixte Processus Infectieux en Milieu Insulaire Tropical, INSERM U1187, CNRS UMR 9192, IRD UMR 249, Université de la Réunion, Plateforme Technologique CYROI, 94791 Sainte Clotilde, La Réunion, France; juliano.haddad@univ-reunion.fr (J.G.H.); victoria.carcauzon@gmail.com (V.C.); omar.el-kalamouni@etudiant.univ-reims.fr (O.E.K.); philippe.despres@univ-reunion.fr (P.D.); 2 UMR QualiSud, Université de La Réunion, CIRAD, Université Montpellier, Montpellier SupAgro, Université d’Avignon, ESIROI, 2 rue J. Wetzell, Parc Technologique Universitaire, 97490 Sainte Clotilde, France; cyrielle.garcia@univ-reunion.fr

**Keywords:** lactic fermentation, *Carica papaya*, antiviral activity, Zika virus

## Abstract

There are a several emerging and re-emerging RNA viruses that are prevalent around the world for which there are no licensed vaccines or antiviral drugs. Zika virus (ZIKV) is an example of an emerging virus that has become a significant concern worldwide because of its association with severe congenital malformations and neurological disorders in adults. Several polyphenol-rich extracts from plants were used as nutraceuticals which exhibit potent in vitro antiviral effects. Here, we demonstrated that the papaya pulp extracted from *Carica papaya* fruit inhibits the infection of ZIKV in human cells without loss of cell viability. At the non-cytotoxic concentrations, papaya pulp extract has the ability to reduce the virus progeny production in ZIKV-infected human cells by at least 4-log, regardless of viral strains tested. Time-of-drug-addition assays revealed that papaya pulp extract interfered with the attachment of viral particles to the host cells. With a view of preserving the properties of papaya pulp over time, lactic fermentation based on the use of bacterial strains *Weissella cibaria* 64, *Lactobacillus plantarum* 75 and *Leuconostoc pseudomesenteroides* 56 was performed and the resulting fermented papaya pulp samples were tested on ZIKV. We found that lactic fermentation of papaya pulp causes a moderate loss of antiviral activity against ZIKV in a bacterial strain-dependent manner. Whereas IC_50_ of the papaya pulp extract was 0.3 mg/mL, we found that fermentation resulted in IC_50_ up to 4 mg/mL. We can conclude that papaya pulp possesses antiviral activity against ZIKV and the fermentation process has a moderate effect on the antiviral effect.

## 1. Introduction

Mosquito-borne Zika virus (ZIKV) belongs to *Flavivirus* genus in the family *Flaviviridae*, which includes viruses at the helm of medically-important tropical diseases such as dengue, West Nile, Japanese encephalitis and yellow fever [1]. ZIKV is transmitted to humans after the bite of an infected *Aedes aegypti* mosquito [2,3,4]. Most of ZIKV strains are members of African or Asian lineage [5], the latter being responsible for contemporary ZIKV epidemics worldwide [6]. Zika disease has become an emerging health threat with significant epidemics in South Pacific, South/Central America and the Caribbean associated to congenital Zika syndrome (CZS) and Guillain-Barre Syndrome in adults [7]. Sexual spread of ZIKV has been reported and the risk for infection through blood transfusion, organ transplantation and perinatal transmission has been documented [6,8,9,10,11,12,13,14].

ZIKV is an enveloped single-stranded RNA virus like other flaviviruses [10]. Viral genomic RNA is initially translated into a single and long polyprotein which is cleaved co- and post-translationally, by cellular and viral proteases, into three structural proteins, C (capsid), prM (membrane) and E (envelope), which comprise the flavivirion, and seven non-structural proteins (NS1, NS2A, NS2B, NS3, NS4A, NS4B and NS5). The NS proteins compose the replication complexes and also contribute to virus subversion towards antiviral innate immunity [15]. ZIKV entry inside the host-cell requires the binding of virions to cellular receptors and their internalization through a clathrin-mediated endocytic pathway [10]. The fusion between the viral and endosomal membranes leads to the release of the viral RNA into the cytoplasm and initiation of the virus replication cycle [16]. Viral RNA replication and assembly of virus particles occur in the environment of the endoplasmic reticulum. Newly assembled viruses are transported through the secretory pathway and infectious virus particles are released into the extracellular compartment [16].

Neither an effective treatment nor a vaccine is available for ZIKV. Given that several countries around the world are at risk of ZIKV outbreaks, it is still of high importance to develop antiviral strategies that include the use of nutraceuticals that can be used to prevent ZIKV infection [17,18,19,20,21]. Several polyphenol-rich extracts from Reunion Island have been reported to prevent ZIKV infection in vitro [22,23,24,25]. The three natural compounds epigallocatechin gallate (EGCG), curcumin and delphinidin exert antiviral effects against ZIKV through a loss of viral infectivity in relation with an inability of virus particles to interact with host-cell receptors [26,27,28]. Polyphenols isoquercitrin, resveratrol and gossypol have also been identified as potent inhibitors of ZIKV entry in the host-cell [21,29,30]. We recently reported that lactic fermentation, using strains of lactic acid bacteria (LAB) isolated from raw fruits and vegetables from Reunion Island, of polyphenol-rich tropical fruit juices or purées such as pineapple, papaya and mango can be a promoting strategy to preserve juices and enhance their antioxidant activity [31,32]. It is suggested that modification in juice polyphenol profile, resulting from lactic acid fermentation, could be linked to the observed increase in radical scavenging activity [33]. Accordingly, a conversion of complex phenolics into free forms and the release of numerous flavonols, anthocyanins and phenolics acids were observed in several fruit and plant fermented juices [34,35,36] that might result in biological activities more relevant to human health in respect to their parent phenolic compounds [37,38]. In this study, we aimed to evaluate the in vitro antiviral activity of three polyphenol-rich tropical fruit juices or pulps, mango, papaya and pineapple, against ZIKV. We further investigated the impact of lactic fermentation, using different LAB, of papaya pulp on its antiviral activity.

## 2. Materials and Methods

### 2.1. Cells, Virus and Reagents

Human alveolar basal epithelial A549 cells (ATCC, CCL-185, Manassas, VA, USA) were cultured in Minimum Essential Medium (MEM: Gibco/Invitrogen, Caslsbad, CA, USA) that contains 5% heat-inactivated Fetal Bovine Serum (FBS, Good purchased from Invitrogen), 1 mmoL/L sodium pyruvate, 2 mmoL/L L-Glutamine, 0.1 mg/mL of streptomycin, 100 U/mL of penicillin and 0.5 µg/mL of fungizone (PAN Biotech, Aidenbach, Germany) at 37 °C and under a 5% CO_2_ atmosphere. The recombinant Zika virus expressing the GFP reporter gene (ZIKV^GFP^) and the clinical isolate PF-25013–18 of ZIKV-PF13 have been previously described [39,40]. The ZIKV progeny production was performed by measuring the quantity of infectious viral particles released into the supernatant by plaque-forming assay on vero cells, as previously described [23]. EGCG was purchased from Sigma-Aldrich.

### 2.2. Juice and Pulp Preparation and Fermentation of Papaya Pulp

Fresh fruits: papaya (*Carica papaya* L. cv. ‘Solo’), pineapple (*Ananas comosus* (L.) Merr. cv. ‘Victoria’) and mango (*Mangifera indica* L. cv. ‘Cogshall’), were obtained from local markets. Fruits were washed, and the peel and seeds were removed from fruits when necessary. For mango and papaya, the pulp was crushed, whereas juice was extracted from pineapple. Juice and pulps were then pasteurized in a water bath for 10 min at 80 °C.

The fermentation of pineapple juice and pulps extracted from mango and papaya fruits was carried out using different bacteria from three species previously isolated from cabbage: *Lactobacillus plantarum* 75, *Weissela cibaria* 64 and *Leuconostoc pseudomesenteroïdes* 56 [32]. Lactic acid bacteria were cultivated in Man Rogosa Sharpe (MRS, Sigma-Aldrich, Saint-Quentin-Fallavier, France) broth at 30 °C. Inocula for food fermentations were prepared by harvesting cells from 24 h cultures in MRS and washing twice in physiological saline water (0.9 % NaCl). Pineapple juice and pulps extracted from mango and papaya fruits were inoculated with cell suspensions, corresponding to 6 log CFU/mL, and incubated at 30 °C for 48 h, then for 7 days at 4 °C. Samples were taken out after 0, 2 and 7 days to determine lactic acid bacteria viability, enumerated by plating on MRS agar. The absence of yeasts and molds was also controlled by plating on glucose chloramphenicol agar medium. Fruit juice and pulps without bacterial inoculum were incubated under the same conditions as a control. All the experiments were performed in triplicate from independent fruit batches. After processing and cold storage, all samples were frozen then freeze-dried for 72 h using a bench-top freeze-drier (Cosmos 80 Cryotec). Stock solutions were prepared from 200 mg of the resulted dry extracts dissolved in 1 mL of sterile PBS and stored at −80 °C until used for the antiviral assays.

### 2.3. Viability Assay

The cellular metabolic activity was evaluated by the MTT (3-[4-dimethylthiazol-2-yl]-2,5-diphenyltetrahzolium bromide) colorimetric assay as described previously [22,23]. A549 epithelial cells were seeded in 96-well plates at a density of 1.5 × 10^4^ cells per well. After 24 h of incubation at 37 °C, the cells were inoculated with the samples at different concentrations (125 to 4000 µg/mL) for 48 h at 37 °C. Next, the supernatants were removed and 20 µL of MTT solution (Sigma-Aldrich, Saint-Quentin-Fallavier, France) at 5 mg/mL was added, and cells were further incubated for 3 h at 37 °C. After incubation, the MTT medium was discard and the formazan crystals were solubilized by adding 50 µL of of Dimethyl sulfoxide (DMSO). Absorbance was measured at 570 nm with a background subtraction at 690 nm.

### 2.4. Flow Cytometry Assay

Cells were washed twice with PBS 1×, harvested with trypsin, fixed with Paraformaldehyde (PFA) 3.7% in PBS for 20 min and then subjected to a flow cytometric analysis using cytoflex (Beckman). Results were analyzed using cytexpert software.

### 2.5. Plaque-Forming Assay

ZIKV growth was titrated and was determined by a plaque-forming assay, as previously described [39]. Vero cells were seeded in a 48-well culture plate and infected with 100 µL of 10-fold dilutions of cell-infected supernatants. After 2 h of incubation at 37 °C, 200 µL of MEM culture medium containing 5% FBS and 0.8% carboxymethylcellulose sodium salt (Sigma-Aldrich, Saint-Quentin-Fallavier, France) were added and infected cells were further incubated for 96 h at 37 °C. Cells were washed with PBS, fixed with PFA 3.7 % in PBS for 20 min and stained with 0.5% crystal violet (Sigma-Aldrich) diluted in 20 % ethanol. Plaques were counted and represented as plaque-forming unit per mL (PFU/mL).

### 2.6. Virus Inactivation Assay

To evaluate the direct effect of papaya pulp extract on viral free particles, ZIKV^GFP^ was incubated with extract for 2 h at 37 °C. The mixture was then subjected to a 50-fold dilution (Multiplicity Of Infection, MOI of 2 PFU/cell) in MEM supplemented with 5% FBS in order to obtain a non-therapeutic concentration of pulp extracts. The diluted mixture was then inoculated on A549 cells cultured in 24-well plates. As a control, ZIKV^GFP^ was mixed with papaya pulp diluted 50-fold and added directly to the cells without an incubation period. The 50-fold dilution served to titrate the plant extract below its effective dose and prevent meaningful interactions with the host cell surface. After 2 h of incubation at 37 °C, the supernatants were removed and replaced with culture medium MEM 5% FBS. The cells were further incubated up to 24 h at 37 °C.

### 2.7. Data Analysis

Statistical analysis consisted of a one-way analysis of variance (ANOVA) followed by Dunnett’s test for multiple comparisons, with a significance of *p* < 0.05. All statistical tests were performed using Prism software (Graphpad version 8.0; La Jola, CA, USA). Degrees of significance are indicated as follows: * *p* < 0.05, ** *p* < 0.01 and *** *p* < 0.001, ns = not significant.

## 3. Results

### 3.1. Papaya Pulp Extract Inhibits ZIKV Infection in Human Cells

**Figure 1:**
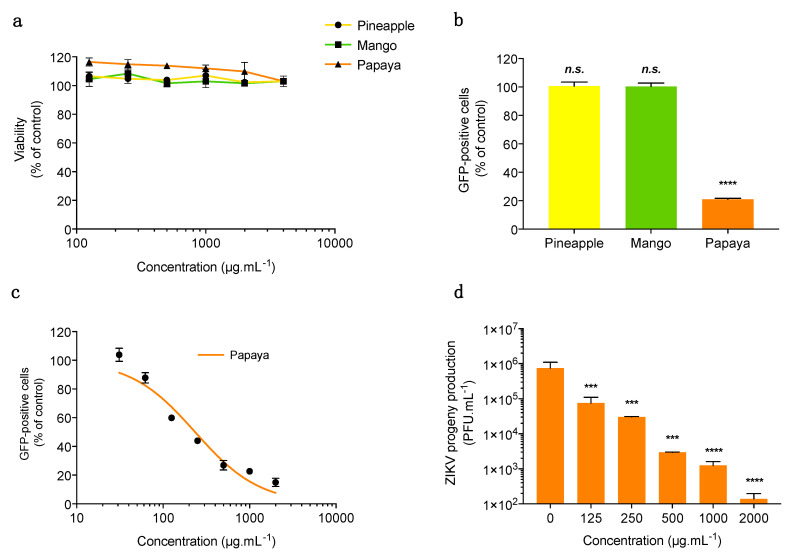
Papaya pulp extract exerts antiviral activity against African and Asian strains of ZIKV in A549 cells. (**a**) A549 cells were incubated with two-fold serial dilutions (125–4000 µg/mL) of pineapple juice, mango or papaya pulp extracts for 48 h. Cell viability was assessed through the MTT assay. Results are means ± standard deviation (SD) of four independent experiments and are expressed as relative value compared to non-treated cells. (**b**) A549 cells were infected with ZIKV^GFP^ at MOI (Multiplicity of Infection) of 2 PFU/cell in the presence of pineapple juice, mango or papaya pulps at 2000 µg/mL. Flow cytometric analysis of GFP fluorescence was performed 24 h post-infection. The results shown are means ± SD of three independent experiments and are expressed as relative value compared to untreated infected cells. (**c**) A549 cells were infected with ZIKV^GFP^ at MOI of 2 PFU/cell and continuously incubated with different concentrations (125–2000 µg/mL) of papaya pulp extract. Flow cytometric analysis of GFP fluorescence was performed 24 h post-infection. The results shown are means ± SD of three independent experiments and are expressed as relative value compared to untreated infected cells. (**d**) A549 cells were infected with ZIKV-PF13 at MOI of 2 PFU/cell and continuously incubated with different concentrations of papaya pulp extract (31–2000 µg/mL). ZIKV progeny production was quantified by the plaque-forming assay. Data represent the means ± SD from four independent experiments. Statistical analyses were performed using a one-way analysis of variance (ANOVA) and Dunnett’s test for multiple comparisons (*** *p* < 0.001, **** *p* < 0.0001; *n.s.* = not significant).

Within a project aiming to investigate the potential antiviral activity of polyphenol-rich juice or pulp of tropical fruit grown on Reunion Island, this study evaluated the potential anti-ZIKV activity of pineapple juice, mango and papaya pulps. After making juice or pulps from the three fruits and prior to evaluating their potential antiviral activity, we determined whether they exhibit cytotoxic effects on human epithelial cell line A549 using the MTT assay (Figure 1a). Cells were treated with different concentrations of juice or pulp extracts ranging from 125 to 4000 µg/mL. Results showed that neither papaya pulp extract nor mango pulp and pineapple juice extracts showed any cytotoxic effects, even at high concentrations (Figure 1a). The concentration of 2000 µg/mL of the three tested fruit juice or pulp extracts was chosen to test their potential antiviral activity.

To evaluate the potential anti-ZIKV activity of pineapple juice, mango and papaya pulps, a chimeric molecular clone of the African ZIKV strain MR766 expressing a GFP reporter gene (ZIKV^GFP^) was used for monitoring viral replication by flow cytometry in A549 cells (Figure 1b). The number of GFP-positive cells decreased around 80% compared to non-treated control cells when 2 mg/mL of papaya pulp extract was added throughout the experiment (Figure 1b). In contrast, treatment of cells with pineapple juice or mango pulp showed no significant effect on ZIKV^GFP^ replication (Figure 1b). Given that mango pulp extract and pineapple juice exert no effect on ZIKV, only papaya pulp extract was studied for its antiviral action against ZIKV.

To assess whether papaya pulp extract inhibits ZIKV infection in a dose-dependent manner, A549 cells were infected for 24 h with ZIKV^GFP^ in the presence of increasing concentrations (20–2000 µg/mL) of papaya pulp extract (Figure 1c). Results showed that papaya pulp extract inhibits ZIKV infection in A549 cells in a dose-dependent manner (Figure 1c). The concentration that inhibited 50 % of GFP-positive cells (IC_50_) was obtained using nonlinear regression following the construction of a sigmoidal concentration-response curve. The IC_50_ was 300 µg/mL for papaya pulp extract (Figure 1c).

We reported that the clinical isolate of ZIKV PF-13 isolated in French Polynesia during the epidemic in 2013 replicates efficiently in A549 cells [39]. To further validate the potential anti-ZIKV effect of papaya pulp extract against the cotemporary epidemic strain, A549 cells were infected for 24 h with ZIKV-PF13 in the presence of increasing concentrations (0–2000 µg/mL) of papaya pulp extract (Figure 1d). A dose-dependent effect of papaya pulp extract on ZIKV growth was observed (Figure 1d). At 1000 µg/mL, papaya pulp extract reduced the virus progeny production by 3-log. Taken together, these data demonstrated for the first time that papaya pulp extract exerts an antiviral effect against both African and Asian ZIKV strains.

### 3.2. Papaya Pulp Extract-Mediated Inhibition of ZIKV Occurs at the Early Stage of the Virus Infectious Cycle

**Figure 2:**
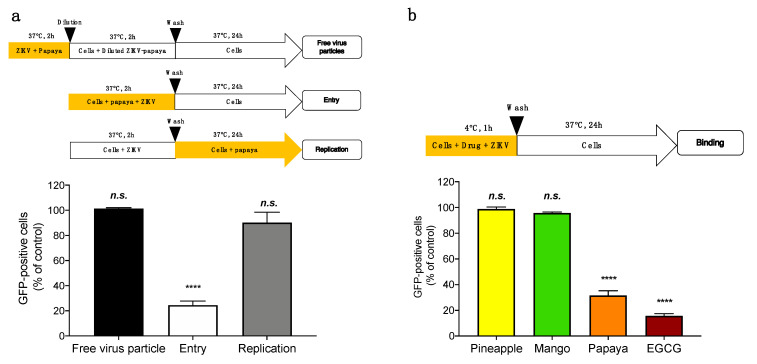
Papaya pulp extract inhibits ZIKV binding to the cell surface. (**a**) Schematic representation of time-of-drug-addition assay used to characterize antiviral activity of papaya pulp extract (1000 µg/mL) on ZIKV^GFP^ infection of A549 cells. Orange arrows indicate the presence of pulp extract during the infection. Flow cytometric analysis of GFP expression in A549 cells infected with ZIKV^GFP^ at MOI of 2 PFU/cell under the experimental conditions shown in the top. (**b**) A549 cells were infected with ZIKV at MOI of 2 PFU/cell for 1 h at 4 °C with or without 1000 µg/mL of papaya pulp extract. Pineapple juice and mango pulp extracts (1000 µg/mL) have been used as negative controls. EGCG (100 µM) was used as a positive control. After 24 h of infection, results of GFP-expression in ZIKV^GFP^-infected A549 cells under the experimental conditions, shown in the top, were analyzed by flow cytometry assay. The data represent the means ± SD of four independent experiments and are expressed as relative values compared to the mock-treated control. Statistical analyses were performed using a one-way ANOVA and Dunnett’s test for multiple comparisons (**** *p* < 0.0001; *n.s.* = not significant).

To further characterize the antiviral mechanism and the stage of ZIKV infection affected by papaya pulp extract, we examined the impact of pulp extract on various stages of the virus replication cycle (free-virus particles, entry and replication). We first investigated whether papaya pulp extract affects the cell-free virions to abolish subsequent infection (Figure 2a, free-virus particles). For this, ZIKV particles were pre-incubated with papaya pulp extract for 2 h at 37 °C and then diluted 50-fold prior to infection. This 50-fold dilution titrates the pulp extract below its effective concentration and prevents interactions of phytocompounds present in the pulp with the cell surface. Flow cytometry assays showed that ZIKV infectivity was not affected by papaya pulp extract. Indeed, results from the virus inactivation assay showed no reduction in virus infectivity when ZIKV^GFP^ was incubated with papaya pulp at 37 °C for 2 h (Figure 2a, free-virus particles). These results indicated that papaya pulp extract does not affect ZIKV particles’ infectivity. Subsequently, time-of-drug-addition assays were performed including different wash steps to ensure the specificity of the treatment on the distinct stage studied and to remove non-meaningful interactions (Figure 2a, schematic representation). Our results showed that papaya pulp extract treatment concomitantly with virus entry severally reduced the percentage of GFP-positive cells (Figure 2a, entry), whereas no significant effect was observed when papaya pulp extract was added after virus entry (Figure 2a, replication). Such results suggest that papaya pulp extract targets the initial stages of infectious life cycle rather than viral replication or viral assembly.

Our data suggest that papaya pulp extract acts by interfering with at least one of the early steps of ZIKV infection. To further elucidate the underlying mechanism of papaya pulp antiviral activity, we subsequently tested its effect on ZIKV entry steps. We investigated whether papaya pulp extract was able to affect viral attachment to the host cells. Thus, binding assays were performed at 4 °C which allows virus binding without viral internalization (Figure 2b). After 1 h of ZIKV binding in the presence of papaya pulp extract, the cells were washed with ice-cold PBS to remove the unbound virus, then shifted to 37 °C without papaya pulp to allow ZIKV internalization (Figure 2b). In parallel, pineapple juice and mango pulp extracts have been used as negative controls. Epigallocatechin gallate, EGCG (100 µM), the main polyphenol compound from green tea, which is known to inhibit ZIKV binding, was used as a positive control [23,27]. Flow cytometry assays showed that the percentage of GFP-positive cells was severally reduced by 75% when the cells are treated with papaya pulp extract as well as the positive control EGCG (Figure 2b). As expected, neither pineapple juice nor mango pulp extract are able to inhibit ZIKV binding to the host cell. These results suggest that papaya pulp extract inhibits ZIKV attachment to A549 cells.

### 3.3. The Lactic Fermentation of Papaya Pulp Affects Its Antiviral Activity Depending on the Bacterial Strain Used during the Fermentation Process

As fresh fruit juices or pulps are rapidly perishable, we recently developed an attractive fermented beverage, through the action of autochthonous lactic acid bacteria isolated from fruit and vegetables grown in Reunion Island, that maintain a pleasant sensory and enhance the antioxidant activity of the tested beverage [31,32]. In this study, we selected three different lactic bacterial strains, *Weissella cibaria* 64, *Leuconostoc pseudomesenteroides* 56 and *Lactobacillus plantarum* 75, to perform lactic fermentation of papaya pulp and aimed to assess whether the lactic fermentation process, using different bacterial strains, could affect the antiviral activity of papaya pulp extract.

The viability of lactic acid bacteria population in fermented papaya pulp reached 12 log CFU/mL after 7 days. Yeast and mold counts were below 1 log CFU/mL in all samples.

**Figure 3:**
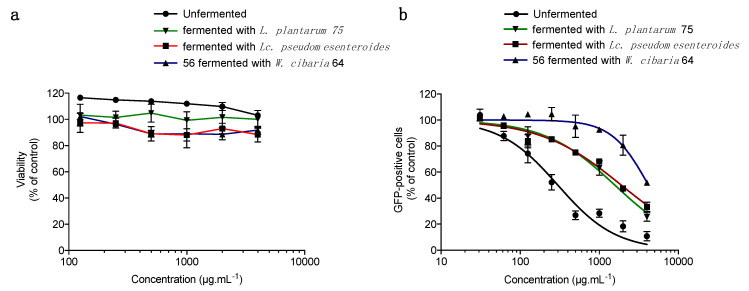
Lactic fermentation of papaya pulp affects its antiviral activity against ZIKV. (**a**) A549 cells were incubated for 48 h with two-fold serial dilutions (125–4000 µg/mL) of extracts of papaya pulp fermented by *L. plantarum* 75, *Lc. pseudomesenteroïdes* 56 or *W. cibaria* 64. Unfermented papaya pulp extract was used as a positive control. Cell viability was assessed through the MTT assay. Results are means ± SD of four independent experiments and are expressed as relative value compared to non-treated cells. (**b**) A549 cells were infected with an input of ZIKV^GFP^ of 2 PFU/cell in the presence of different concentrations (31–4000 µg/mL) of fermented papaya pulp extracts. Unfermented papaya pulp extract was used as a positive control. Flow cytometric analysis of GFP fluorescence was performed 24 h post-infection. The results shown are means ± SD of three independent experiments and are expressed as relative value compared to untreated infected cells.

Prior to evaluating the antiviral activity of fermented pulp extracts, we measured the cytotoxic effect (Figure 3a) of the fermented pulp extracts obtained through the fermentation with each of the three bacterial strains, *W. cibaria* 64, *Lc. pseudomesenteroides* 56 and *L. plantarum* 75. The unfermented pulp was used as a positive control. Plotting cell viability against different concentrations (125–4000 µg/mL) of the three fermented pulp extracts revealed that all fermented extracts do not show any cytotoxic effect on A549 cells as well as the unfermented papaya pulp extract (Figure 3a).

To assess the antiviral activity of the three fermented papaya pulps against ZIKV, A549 cells were infected with ZIKV^GFP^ and continuously treated with different concentrations of the fermented papaya pulp extracts (Figure 3b). The unfermented papaya pulp extract was used as a positive control. Our data showed that all three fermented papaya pulp extracts showed a dose-dependent antiviral activity against ZIKV (Figure 3b). The concentration that inhibited 50% (IC_50_) of ZIKV infection was obtained using nonlinear regression, following the construction of a sigmoidal concentrations–responses curve (Figure 3b). As shown above, the IC_50_ of the unfermented papaya pulp extract was 0.3 mg/mL, whereas the IC_50_ of papaya pulp extracts fermented with *L. plantarum*, *Lc. pseudomesenteroïdes* and *W. cibaria* were 1.5, 1.9 and 4.2 mg/mL, respectively. Interestingly, our data show that the lactic fermentation of papaya pulp affects its antiviral activity against ZIKV depending of the lactic bacterial strain used. The fermentation of papaya pulp by *L. plantarum* or *Lc. pseudomesenteroïdes* reduces the antiviral activity of papaya pulp by 5- to 6-fold compared to unfermented papaya pulp. But fermentation with *W. cibaria* 64 reduces the antiviral activity of papaya pulp by nearly 14-fold.

### 3.4. Concluding Remarks

The development of plant-derived substances used as nutraceuticals and capable of inhibiting the infection of emerging viruses represents an attractive and eco-friendly strategy [41]. Indeed, several studies have shown that plants represent a promising source of antivirals against emerging viruses [17,18,19,22,23,24,30,42,43]. In this study, we showed for the first time that papaya fruit pulp inhibited the infection of A549 cells by the epidemic ZIKV without reducing cell viability. In addition, our results suggest that the papaya pulp extract inhibits the attachment of ZIKV to A549 cells, thus preventing the viral particles from initiating an infectious cycle.

It has been reported that carpine, a phytochemical identified in alkaloidal fraction from the leaves of *Carica papaya*, commonly known as papaya, exhibit potential in vitro antiviral activity against dengue virus (DENV), a closely related flavivirus [44]. However, the mechanism of antiviral action against DENV has not yet been elucidated.

Moreover, the outcomes presented in this study underscore the impact of the lactic fermentation of papaya fruit pulp on its antiviral activity. We, as of late, reported that the lactic fermentation could enhance the antioxidant activity of papaya juice [31]. Our data showed that the lactic fermentation diminishes the antiviral efficacy of papaya pulp, when compared to the unfermented one, contingent upon the lactic bacterium used. These changes of antiviral activity related to the bacterial strains probably reflect different enzymatic activities that can modulate the phenolic composition of fruit pulp over fermentation [34,35]. Further investigations of changes in papaya pulp phytochemical composition during lactic fermentation with different bacterial strains associated with enzymatic activities, involved in the hydrolysis of polyphenolic compounds, should provide better insight. It would be of great interest to determine which component(s) are responsible for the antiviral activity or whether there is a synergetic effect of several compounds leading to anti-ZIKV activity.

## 4. Conclusions

In conclusion, the present study reinforced the importance of papaya fruit pulp as a potent flavivirus inhibitor acting on viral infectivity. It will now be of great interest to determine whether papaya pulp extract and its resulting fermented pulp exert antiviral effect against other medically important flaviviruses, with the four serotypes of dengue virus as a priority.
